# Parental External Locus of Control in Pregnancy Is Associated with Subsequent Teacher Ratings of Negative Behavior in Primary School: Findings from a British Birth Cohort

**DOI:** 10.3389/fpsyg.2018.00120

**Published:** 2018-02-09

**Authors:** Stephen Nowicki, Steven Gregory, Genette L. Ellis, Yasmin Iles-Caven, Jean Golding

**Affiliations:** ^1^Department of Psychology, Emory University, Atlanta, GA, United States; ^2^Centre for Child and Adolescent Health, Bristol Medical School, University of Bristol, Bristol, United Kingdom

**Keywords:** ALSPAC, parental locus of control, child behavior, SDQ, teacher assessment

## Abstract

The purpose of the present study was to examine whether parents’ locus of control (LOC) obtained before the birth of their child predicts the child’s behavior at school in School Years 3 (ages 7–8) and 6 (ages 10–11). A modified version of the adult Nowicki–Strickland internal–external locus of control scale was completed by mothers and fathers in their own home during pregnancy. Externality was defined as a score greater than the median and internality as equal to, or less than, the median. Outcomes were the five individual subscales and the total difficulties of Goodman’s strengths and difficulties’ questionnaire completed by the children’s class teachers at the end of School Years 3 and 6. As predicted, it was found that the greater the presence of externality in the parents, the greater the increased risk of the child’s adverse behavior as rated by teachers. The risk was generally greatest if both parents were external and lowest if both were internal. There was a consistent relationship at both Year 3 and Year 6 between maternal externality in pregnancy and children’s emotional difficulties. However, for other behaviors, the pattern of associations varied depending on whether the mother or father was external, the type of adverse behavior, and the School Year in which children were assessed. Prenatal parental externality appears to be significantly associated with a variety of children’s negative behaviors. Of note was the finding that fathers’ as well as mothers’ LOC was important in determining children’s outcomes. Implications of the complexity of the results for the role parents may play in children’s personality and adjustment are discussed.

## Introduction

Most researchers believe that parents have a significant impact on their children’s personality and behavior. Baumrind (1991) identified three parental styles, authoritative, authoritarian, and permissive and suggested that each was associated with different child outcomes. Generally, authoritative parenting is characterized by high warmth and responsiveness (e.g., Supple and Small, 2006). Authoritative parents establish clear rules for their children and give reasons for their expectations (e.g., Carlo et al., 2007). In contrast, authoritarian parents exhibit high levels of controlling behavior and low levels of responsiveness (e.g., Luyckx et al., 2007) while permissive parents are high in responsiveness and low in demandingness. Research suggests that not only do negative parenting styles, such as excessive harshness or laxity, predict negative child outcomes, such as anxiety or externalizing behavior (e.g., Bayer et al., 2008), but positive parenting styles, marked by warmth and realistic boundaries, relate to positive child outcomes, such as empathy or prosocial behavior (e.g., Davidov and Grusec, 2006).

Although not studied as frequently as parental styles, parent personality may also be significantly associated with children’s outcomes. By virtue of its association with behavioral outcomes, one possible parental variable is LOC. Rotter (1966) introduced the concept of LOC and defined it as a generalized problem solving expectancy as follows: “Internal versus external control refers to the degree to which persons expect that a reinforcement or an outcome of their behavior is contingent on their own behavior or personal characteristics versus the degree to which persons expect that the reinforcement or outcome is a function of chance, luck, or fate, is under the control of powerful others, or is simply unpredictable. Such expectancies may generalize along a gradient based on the degree of semantic similarity of the situational cues.”

His article stimulated a remarkable amount of research involving the LOC construct for the next half century. Using a variety of LOC measures, investigators have published over 17,000 studies on this topic (Nowicki and Duke, 2016). Although many types of scales have been used to assess LOC, significant findings have been replicated across an impressive variety of psychological outcomes with internality such as improved academic achievement (e.g., Kalechstein and Nowicki, 1997; Flouri, 2006), sports performance (e.g., Arnaud and Palazzolo, 2012), and business success (e.g., Spector et al., 2002; Wu et al., 2015).

In one of the earliest reviews of the locus of control/personality/behavior relationships, Strickland (1978) concluded that externality, like parenting style, was “linked with pathological difficulties for both children and adults.” Since that review, additional studies have provided results that support associations between external LOC and negative attitudes, personality characteristics, and behavior in both adults and children. For example, external LOC in *adults* has been associated with a variety of negative personality characteristics (e.g., Nowicki and Duke, 1974; Wheeler and White, 1991), depression (Benassi et al., 1988; Christensen et al., 1991; Bjørkløf et al., 2013), anxiety (Richert, 1981; Carden et al., 2004), and psychoses (Harrow et al., 2009; Weintraub et al., 2016).

Likewise, external LOC has been correlated with an impressive variety of negative personal and social outcomes in *children* (Nowicki and Duke, 1983; Nowicki, 2016, Manual for the Nowicki-Strickland internal external scales, Unpublished). Higher externality is related to increased chances of being: sexually abused (Beech and Ford, 2006), suicidal (Liu et al., 2005), depressed (Benassi et al., 1988; Luthar and Blatt, 1993), enuretic (Butler, 2001), learning disabled (Dudley-Marling et al., 1982), as well as having lower self-esteem (Wickline et al., 2011), attention deficit disorder (Ialongo et al., 1993), and more trouble persisting (McLeod, 1985). However, because most past studies are cross-sectional in design, cause and effect cannot be ascertained.

To further clarify the association between parental LOC and child outcome, one group of researchers has focused on the specific *parenting* LOC and not a global LOC in parents and its possible relationship with child behavior (Campis et al., 1986). Investigators have found that specific parenting externality in one or both parents was associated with negative outcomes in preschool (e.g., Estroff et al., 1994), preadolescent, and adolescent participants (e.g., Freed and Tompson, 2011), as well as a greater likelihood of receiving diagnoses of attention deficit/hyperactivity (Hoza et al., 2000) or anxiety (Becker et al., 2010). In addition, Moreland et al. (2016) found parenting externality to be associated with a greater likelihood of children being disruptive and less able to “cope,” although when parenting LOC became more internal, their children’s behavior also became less negative.

The possible connection between both global LOC and specific parenting LOC with children’s outcomes is further supported by the results of other researchers. For example, Hagekull et al. (2001) found that greater parenting externality measured when children were 33 months and at 9 years of age was related to greater child difficulties both concurrently and prospectively. They concluded that the results pointed to “parents’ perceived control as important for their children’s development of externalizing and internalizing problems as well as for social and non-social competence development,” and having an independent impact on development during the preschool years over and above infant temperament and acting out behavior.

While it is apparent that both generalized and specific parenting LOC are associated with a variety of child outcomes, fewer studies have examined how global *prenatal* parents’ LOC is related to children’s outcomes beginning soon after birth and through pre-adolescence. Because most past studies have gathered parent LOC and child behavior simultaneously, it makes the task of separating out who is affecting whom even more difficult. However, while not implying causation, having parent LOC obtained before it is affected by interactions with the child following birth can provide information about the parent LOC, child outcome association, not previously available. In an earlier set of analyses (Nowicki et al., 2017), we found that a generalized prenatal parent LOC predicted preschool children’s personality and social behavior during the 5 years after their birth. We used data gleaned from ALSPAC, a cohort study which has been gathering data from parents and their children beginning during pregnancy in 1990–1992 (see section “Materials and Methods”) to the present day. We found that prenatal parent LOC predicted child eating, sleeping, and anger management outcomes from birth to 5 years of life; the greater the presence of parent externality, the greater the likelihood of negative child outcomes as reported by the mother (Nowicki et al., 2017).

The purpose of this paper was to evaluate the association between prenatal parent LOC and school-age children’s personal and social behavior within the school environment. Teachers, unaware of the LOC of the parents, were asked to rate children’s behavior based on what they observed in school. We aimed to ascertain whether prenatal parent externality continued to be associated with children’s negative behavior in the school situation where they were not evaluated by family members, but by others (teachers) who observed them outside the home.

More specifically, the following predictions were made.

(1) Social learning theory (Rotter, 1954) and past empirical research (e.g., Lefcourt, 1982) suggest that the more external parents are the less likely they are to be organized, persistent, and responsible compared to their more internal peers. Because of these characteristics, external parents may be less able to solve child-raising problems with the result that their children will be more likely to have negative personality characteristics and behavioral difficulties. Therefore, it is predicted that the prenatal parent externality association with behavioral difficulties previously found in children up to age 5 will also be found at Year 3 (7–8 years of age) and Year 6 (10–11 years of age).

(2) Ollendick (1979) administered parents a generalized LOC scale, similar to the one used in the present study, to each parent. To analyze his findings, he created four combinations of parent LOC: both mother and father internal, father internal, mother external; mother internal, father external; both mother and father external. Ollendick predicted and found that the presence of externality in at least one parent, especially the mother, was associated with more negative child outcomes when compared with both parents being internal. We used a similar design and predict a similar result in the present study.

## Materials and Methods

### Participants

The ALSPAC pre-birth cohort was designed to determine the environmental and genetic factors that are associated with health and development of the study offspring (Golding and ALSPAC Study Team, 2004; Boyd et al., 2013). As part of the study design, therefore, there was a concerted effort before the child’s birth to obtain from the parents details of their own personalities, moods, and attitudes, including a measure of their LOC.

ALSPAC recruited 14,541 pregnant women who resided in Avon, United Kingdom, with expected dates of delivery between 1st April 1991 and 31st December 1992 (an estimated 80% of the eligible population). Data were collected at various time-points using self-completion questionnaires, biological samples, hands-on measurements, and linkage to other data sets. With the advice of the ALSPAC Ethics and Law Advisory Committee, it was decided not to enroll the study fathers directly, but rather to send to the mother a questionnaire for her partner and ask her if she would like her partner to be involved, and if so whether she would be good enough to pass the questionnaire on with a separate reply-paid envelope for return. The study deliberately had no information on whether the mother had invited her partner to take part except when the completed questionnaire was returned. It should be noted that in consequence of this format, there was no way in which the study could send reminders to the partners themselves. In the event, at least one questionnaire was returned by 75% of the partners of women who were taking part in the study. The ALSPAC Ethics and Law Advisory Committee agreed that consent was implied if questionnaires were returned. Informed written consent was obtained for all biological samples prior to analysis and for certain invasive procedures during the hands-on assessments (which were optional to attend).

For this project, we have concentrated on the data collected from questionnaires completed by both the mother and her partner *before* the birth of the study child. The information on the child’s behavior was obtained using a self-completion questionnaire completed by the child’s teacher at the end of School Years 3 (ages 7–8) and 6 (ages 10–11). The study website contains details of all the data that are available through a fully searchable data dictionary: www.bristol.ac.uk/alspac/researchers/data-access/data-dictionary/.

Ethical approval for the study was obtained from the ALSPAC Ethics and Law Committee and the Local Research Ethics Committees.

### Exposure Measure: Locus of Control

The ANSIE (Nowicki and Duke, 1974) followed Rotter’s definition in its construction. It has an easier reading level than the Rotter scale, and is significantly correlated with Rotter’s test (Nowicki,2016, unpublished) making it appropriate for testing adults from the general population.

An anglicized and briefer form of the ANSIE was used in the present study. It contained the 12 items from the original 40 item scale which possessed the highest item-total correlations based on the responses of 135 mothers. The scales were completed by each parent at home in mid-pregnancy. Factor analysis of responses from 12,471 women confirmed the single factor structure of the scale. Coefficient alpha was 0.78 in this population. The scores ranged from 0 to 12 and were roughly normally distributed with medians of 4 and 3 for the mothers (*n* = 12,471) and their partners (*n* = 8,645) respectively. The higher the score, the more external the LOC. As in our previous publications, external LOC was defined as above the median while internal LOC was defined as scores equal to or lower than the median (Golding et al., 2017a,b,c; Nowicki et al., 2017). The median score for the mother was 4, and for the father, it was 3.

### Child Outcomes: Strengths and Difficulties’ Questionnaire

Class teachers completed the teacher version of the SDQ (Goodman, 1997), a widely used measure of child and adolescent mental health. This was administered toward the end of the school year (June–July) for study pupils in School Years 3 and 6 (when the children were aged approximately 7–8 and 10–11 years old, respectively). All primary schools in the study area were approached, and for the children who had moved out of the area, parents were sent the questionnaire to give to the teacher.

The questionnaire measures five mental health constructs under investigation in this study: attention difficulty/ hyperactivity, conduct problems, emotional symptoms, peer difficulties, and prosocial behavior. Each construct is measured with five items rated on a three-point Likert scale (0 – not true; 1 – somewhat true; or 2 – certainly true). Total scores for each construct range from 0 to 10, with higher scores indicating more severe problems, but greater (better) levels of prosocial behavior. A total difficulties score comprised the sum of the scores for each behavior except the prosocial score. When an item was not completed in a scale, it was prorated – i.e., replaced with the average for the other items in the scale for that child. Internal consistency across the different constructs of the SDQ and across different informants (self-report, teacher, and parent) has been found to be satisfactory (Cronbach’s alpha mean of 0.73). Test–retest stability after 4–6 months has been reported to be 0.62 (Goodman, 2001).

### Other Variables Considered

In order to assess the different confounders measured in pregnancy that could have influenced the results, we considered the following: maternal age (defined as her age at the last menstrual period prior to conception of the study child); parity (the number of previous pregnancies resulting in either a live or stillbirth); the housing situation [owner/occupied; council (public) housing; other rented]; crowding (ratio of the number of persons in the home divided by the number of rooms – excluding bathrooms and small kitchens); whether or not the woman was smoking in mid-pregnancy; whether she had had one or more days of binge drinking (4+ units of alcohol); maternal education (the highest educational achievements – 3 levels); whether she reported difficulty in affording to buy food; and whether she was depressed in mid-pregnancy (score of 12+ on the EPDS measure) (Cox et al., 1987).

### Statistical Methodology

In this study, we explored the associations between the study child’s behavior as reported by the teacher and the externality/internality of the parents. We compared the child’s behavior outcomes using both the mean behavior scores (using multiple regression) and the risk of adverse behavior measured as the worst ∼10% of the score (using logistic regression). In order to distinguish between the risks of adverse behavior contributed by different numbers of external parents within the family, a derived variable concerning the number of such parents was used, and the results presented as odds’ ratios with 95% confidence interval.

## Results

### Bias in Response

The response rate of the primary school teachers of the study children had the advantage of not being biased by the social circumstances of the study families. Of the 12,471 children whose mothers had completed the LOC measure in pregnancy, there were 5660 (40%) and 6492 (52%) who had teacher reported SDQ scores in Years 3 and 6, respectively. Although no statistically significant differences were found between those circumstances of parents whose teachers responded compared with those who did not in regard of parity, difficulty affording food, binge drinking in pregnancy, maternal prenatal depression, or maternal LOC, there was a difference in the prevalence of young mothers and those living in rented housing (Supplementary Table 1). In addition, although attributes such as overcrowding, maternal smoking, and maternal education level differed for one of the two assessments, absolute differences were small.

### The Child’s Behavior and Each Parents’ Individual LOC Orientations

The way in which the differing behavior scores and the parental LOC scores correlate is shown in Supplementary Table 2. The contemporaneous LOC comparisons were medium in size (*r* = 0.32), but the correlations between each LOC score and their children’s behaviors were only small. However, such minimal results were detected only when the LOC scales were treated as continuous. When a dichotomy was used to distinguish externally oriented individuals from the rest of the population, a much clearer pattern was shown. Mothers who were external were more likely to have children judged negatively by the teachers on all scales of the SDQ at the end of Year 3, although prosocial behavior and peer problems scales had a much weaker association at the end of Year 6. In contrast, for the children of externally oriented fathers, relationships were minimal in Year 3 but were significant at 0.002 or lower in Year 6 (**Table 1**). In order to assess whether these differences were mirrored in lifestyle patterns, we charted the differences between the four groups (Supplementary Table 3). These conformed to the findings that external individuals were less likely to have obtained educational qualifications, but more likely to have babies earlier, smoke, binge drink, and be depressed in pregnancy.

**Table 1:** Mean (SD) of child behaviors (unadjusted) as assessed by the child’s teacher according to maternal and paternal LOC as measured in pregnancy. (The higher the prosocial score, the better the behavior, but for all other scales, the higher the score, the worse the behavior.)

SDQ	Mother external		Mother internal	Father external		Father internal
*Year 3 (ages 7–8)*	*N* ∼ 5656			*N* ∼ 4002		
Prosocial	7.57 (2.47)		7.86 (2.38)	7.76 (2.35)		7.86 (2.37)
		*P* < 0.0001			*P* = 0.152	
Hyperactivity	2.97 (2.78)		2.29 (2.51)	2.72 (2.71)		2.24 (2.50)
		*P* < 0.0001			*P* < 0.0001	
Emotional difficulties	1.53 (2.06)		1.25 (1.83)	1.43 (1.97)		1.27 (1.84)
		*P* < 0.0001			*P* = 0.008	
Conduct problems	0.89 (1.60)		0.59 (1.23)	0.75 (1.46)		0.59 (1.22)
		*P* < 0.0001			*P* < 0.001	
Peer difficulties	1.27 (1.82)		1.07 (1.70)	1.18 (1.78)		1.09 (1.72)
		*P* < 0.0001			*P* = 0.138	
Total behavioral difficulties	6.66 (5.99)		5.19 (5.20)	6.07 (5.71)		5.19 (5.24)
		*P* < 0.0001			*P* < 0.0001	
*Year 6 (ages 10–11)*	*N* ∼ 6500		*N* ∼ 4527			
Prosocial	7.82 (2.42)		7.97 (2.34)	7.85 (2.44)		8.13 (2.28)
		*P* = 0.016			*P* < 0.0001	
Hyperactivity	2.56 (2.73)		1.98 (2.49)	2.49 (2.74)		1.77 (2.33)
		*P* < 0.0001			*P* < 0.0001	
Emotional difficulties	1.38 (1.94)		1.19 (1.77)	1.37 (1.92)		1.18 (1.77)
		*P* < 0.0001			*P* < 0.001	
Conduct problems	0.96 (1.70)		0.68 (1.40)	0.92 (1.67)		0.58 (1.27)
		*P* < 0.0001			*P* < 0.0001	
Peer problems	1.25 (1.86)		1.17 (1.82)	1.27 (1.91)		1.10 (1.77)
		*P* = 0.072			*P* = 0.002	
Total behavioral difficulties	6.15 (5.94)		5.01 (5.50)	6.05 (6.10)		4.63 (5.16)
		*P* < 0.0001			*P* < 0.0001	

### The Child’s Behavior and the Internality and/or Externality of the Pairs of Parents

In **Table 2**, the mean behavior scores of the children are shown for the four combinations of parents: both external; mother external, father internal; mother internal, father external; and both internal.

**Table 2:** Mean (SD) of teacher ratings of child behavior using SDQ according to the LOC orientation of the child’s parents as measured in pregnancy.

Child behavior	M.Ex. F.Ex	M.Ex. F.In	M.In. F.Ex	M.In. F.In
Hyperactivity				
Year 3	2.99 [2.79]	2.57 [2.66]^∗∗^	2.40 [2.58]	2.07 [2.40]^∗∗^
Year 6	2.74 [2.84]	2.00 [2.39]^∗∗∗^	2.16 [2.56]^a^	1.66 [2.28]^∗∗∗^
Emotional problems				
Year 3	1.53 [2.05]	1.43 [2.01]	1.30 [1.85]	1.19 [1.74]
Year 6	1.44 [1.97]	1.28 [1.92]	1.28 [1.84]	1.13 [1.70]^∗^
Conduct problems				
Year 3	0.87 [1.61]	0.72 [1.38]^∗^	0.59 [1.26]	0.53 [1.13]
Year 6	1.02 [1.74]	0.61 [1.26]^∗∗∗^	0.77 [1.55]^a^	0.56 [1.27]^∗∗∗^
Peer problems				
Year 3	1.26 [1.81]	1.19 [1.81]	1.06 [1.72]	1.04 [1.67]
Year 6	1.28 [1.90]	1.07 [1.71]^∗^	1.25 [1.93]	1.11 [1.80]
Total difficulties				
Year 3	6.66 [5.97]	5.91 [5.59]^∗∗^	5.34 [5.26]	4.83 [5.01]^∗^
Year 6	6.47 [6.23]	4.96 [5.01]^∗∗∗^	5.46 [5.85]^a^	4.46 [5.18]^∗∗∗^

*^a^Difference between M.Ex.F.In and M.In.F.Ex: P < 0.05. (The higher the score, the worse the behavior.) [Asterisks indicate differences between the pairs of father orientation as ^∗^P < 0.05; ^∗∗^P < 0.01; ^∗∗∗^P < 0.001.]*

In every case in which both parents were external, their children had a greater number of teacher rated difficulties compared to children who had any other combination of parent LOC. The differences were significant in all cases in which children where both parents were external were compared to the children both of whose parents were internal. In general, the mean behavior difficulties scores where one parent was internally and one externally oriented were midway between the scores where both parents had an internal and both an external LOC. This was illustrated further by the relationship found between the risk of the child having a behavior difficulty score in the worst 10–15% of scores and the number of parents who had an external LOC (**Table 3**). Unadjusted data show the risk of adverse behavior for each extra external parent. In general, except for prosocial behavior in School Year 3 and peer problems at both time points, there were significant increases in risk for each extra external parent. The greatest change in risk was found for hyperactivity in Year 6 (with an increased risk of 61% per increase in one external parent), and conduct problems at both time points (increase of approximately 50%). Adjustment reduced the odds’ ratios to a certain extent, but only for emotional symptoms were the significances of the relationships considerably reduced.

**Table 3:** The change in the odds of the child’s adverse behavior according to the numbers of parents who have an external orientation during pregnancy [odds are per increase in one external parent].

Child behavior	Unadjusted		Adjusted^a^	
	OR [95% CI]	*P*	OR [95% CI]	*P*
Prosocial				
Year 3	1.04 [0.91,1.19]	0.559	1.00 [0.86,1.16]	0.962
Year 6	**1.24 [1.09,1.40]**	**0.001**	**1.23 [1.08,1.41]**	**0.003**
Hyperactivity				
Year 3	**1.40 [1.25,1.57]**	**<0.001**	**1.32 [1.16,1.50]**	**<0.001**
Year 6	**1.61 [1.43,1.81]**	**<0.001**	**1.49 [1.30,1.70]**	**<0.001**
Emotional symptoms				
Year 3	**1.26 [1.12,1.41]**	**<0.001**	**1.18 [1.04,1.34]**	**0.011**
Year 6	**1.20 [1.08,1.35]**	**0.001**	1.12 [0.99,1.26]	0.079
Conduct problems				
Year 3	**1.50 [1.30,1.73]**	**<0.001**	**1.32 [1.12,1.54]**	**0.001**
Year 6	**1.52 [1.34,1.72]**	**<0.001**	**1.37 [1.19,1.57]**	**<0.001**
Peer problems				
Year 3	1.09 [0.96,1.24]	0.185	1.09 [0.95,1.14]	0.615
Year 6	1.07 [0.95,1.21]	0.234	1.05 [0.92,1.19]	0.449
Total difficulties				
Year 3	**1.45 [1.28,1.64]**	**<0.001**	**1.34 [1.17,1.54]**	**<0.001**
Year 6	**1.39 [1.24,1.56]**	**<0.001**	**1.25 [1.10,1.43]**	**0.001**

*Bold values are statistically significant at *P* < 0.05. ^a^Adjusted for maternal age, residence in public rented housing, and child’s sex.*

In order to examine whether Ollendick’s (1979) prediction that mothers’ as opposed to fathers’ internality would have a more positive impact on child outcomes, we selected parent combinations in which either the mother or the father was external, while their corresponding parent was internal. As shown in **Table 4**, this prediction was supported by findings at Year 3, where children were similar in age to Ollendick’s; internal mothers paired with external fathers had children whose teachers rated them more favorably on all the specific SDQ behaviors as well as on the total difficulties scale compared to external mothers and internal fathers, although none of the differences were statistically significant. However, somewhat surprisingly, this relationship was reversed when examining Year 6 children who were considerably older than those used by Ollendick. Here, except for emotional problems, external fathers paired with internal mothers were more likely to have children with more difficulties compared to external mothers with internal fathers. Examination of the proportions of children with the worst behavior scores revealed a similar pattern (Supplementary Table 4).

**Table 4:** Comparison between the odds of the child’s adverse behavior when just one parent is externally oriented during pregnancy [odds are for difference if it is the mother who is external].

Child behavior	Unadjusted		Adjusted^a^	
	OR [95% CI]	*P*	OR [95% CI]	*P*
Prosocial				
Year 3	1.22 [0.87,1.71]	0.254	1.20 [0.85,1.71]	0.299
Year 6	0.82 [0.59,1.13]	0.215	0.82 [0.58,1.14]	0.240
Hyperactivity				
Year 3	1.12 [0.83,1.51]	0.452	1.12 [0.83,1.54]	0.446
Year 6	0.84 [0.61,1.14]	0.257	0.89 [0.64,1.24]	0.497
Emotional symptoms				
Year 3	1.20 [0.90,1.61]	0.222	1.23 [0.91,1.65]	0.174
Year 6	1.04 [0.78,1.39]	0.797	1.03 [0.76,1.38]	0.868
Conduct problems				
Year 3	1.15 [0.80,1.66]	0.453	1.11 [0.76,1.63]	0.588
Year 6	**0.68 [0.49,0.95]**	**0.026**	0.75 [0.53,1.06]	0.103
Peer problems				
Year 3	1.20 [0.87,1.66]	0.263	1.21 [0.87,1.68]	0.255
Year 6	**0.67 [0.49,0.92]**	**0.012**	**0.67 [0.49,0.93]**	**0.015**
Total difficulties				
Year 3	1.32 [0.96,1.81]	0.084	1.31 [0.95,1.81]	0.105
Year 6	0.81 [0.59,1.11]	0.194	0.86 [0.62,1.19]	0.369

*Bold values are statistically significant at P < 0.05. ^a^Adjusted for maternal age, residence in public rented housing, and child’s sex.*

When individual SDQ scales are examined, all mean differences are in the direction of showing that the more parents that were external, the larger the increase in child’s behavior difficulties. After adjustment, the effect sizes reduced slightly but almost all were statistically significant with the exception of prosocial behavior in Year 3 and peer problems in Year 6 (Supplementary Table 5). The findings suggest that prenatal parent externality is associated with a greater number of negative children’s outcomes, depending on the developmental stage of the children.

These findings suggest the intriguing possibility that mothers’ internality may be more important when children are younger than older and that fathers’ LOC may be more likely than mothers’ to be associated with children’s behavior when they approach adolescence.

## Discussion

The greater the presence of externality in parents prenatally, the greater the number of teacher rated difficulties in their children approximately 8 and 11 years later. Children’s behavioral outcomes were not assessed by their parents, but by independent observers (their teachers) with no knowledge of parents’ prenatal LOC orientation. Putting the present study’s findings together with those of an earlier one (Nowicki et al., 2017) suggests that the association between prenatal parental externality and negative children’s behavior is present soon after birth in the childhood home and continues to be present when children attend full time school, at least up to preadolescence.

The present study examined the association not only between completely internal or external parent combinations and child outcomes but also between parent dyads in which one parent was internal and the other external and child outcomes. Using the four combinations of prenatal parent LOC allowed for the evaluation of the contribution of each parent’s LOC orientation to the association with children’s outcomes. In most cases, it did not appear to matter whether the mother or the father was the source of parent externality, the result was the same; externality was associated with negative child outcomes. Although girls had fewer absolute numbers of teacher rated difficulties than boys, they showed a similar pattern of prenatal parent externality associated with negative outcomes.

The results are comparable to those obtained during the first 5 years of life (Nowicki et al., 2017). Children of parents who were both prenatally externally controlled experienced more difficulties in sleeping, eating, and dealing with anger than their peers who had parents who were both prenatally internally controlled. However, unlike in the present study, the child outcomes and interactions took place within the home and parents were responsible for reporting on their child’s outcomes.

What is it about parental externality that may translate into how they interact with their children? Campis et al. (1986) suggest that parents “with external parental LOC orientations possess several negative concomitant attitudes about their parental roles such as low self-efficacy and a sense of being dominated by their child’s demands.” Lefcourt (1982) agrees. Using the assumptions of Rotter’s (1954, 1966) social learning theory, Lefcourt reasoned not only that externally controlled parents would tend to see their children’s behavior as being outside their own efforts, but because they saw themselves as relatively powerless they would be inconsistent in setting limits for their children’s activities. This lack of structure, consistency, and limits could create problems for children attempting to learn, through feedback, how to behave appropriately.

One possible way of affecting the parent externality/child negative behavior association would be to provide ways for parents to learn to be more internal. Support for this possibility comes from Hagekull et al. (2001) who found that changes toward parenting internality was also associated with fewer indications of behavioral problems in their children. It remains to be seen if changes in a more generalized LOC would have a corresponding effect on children’s rated difficulties.

Perhaps what schools could do to help children coming from families with prenatally external parents is to provide children with the kinds of learning experiences and structures to help them become aware of, and to learn from, the connections that exist between how they behave and what happens to them. Such experiences may help children develop appropriate levels of internal control; such changes may function to neutralize the possible negative impact of parental externality. If, on the other hand, that kind of intervention is beyond the resources of the schools, teachers could modify their teaching methods to provide more structure for children coming from the unstructured environments provided by externally controlled parents. Such interventions have proved to be successful with externally controlled children who have been found to respond more effectively to structure and primary reinforcement than their internal peers (Nowicki, 2016).

### Limitations

Although previous researchers have found authoritative parental style related to children’s internality and authoritarian parental style to children’s externality (e.g., Wickline et al., 2011), they have failed to identify parental practices that may connect parental style and LOC with children’s outcomes. Darling and Steinberg (1993) were among the first to suggest that it is parental practices and not parenting styles that have a more direct impact on children’s outcomes. While past studies have had some success identifying parent behaviors associated with children’s LOC orientations (Carton and Nowicki, 1994; Carton et al., 1996), there is a lack of information about actual parental practices and behaviors shown by different parenting styles in relation to their LOC orientations.

Because cause and effect could not be established in the present study, it is not only possible, but probable that parent and child temperament, cognitive ability, and the like would affect the parent LOC/child outcome association. Future researchers should investigate the role of these and other personality and cognitive factors, as well as seeking to identify parent and child behaviors that characterized parent dyads differing in externality especially when parents and children are interacting with one another.

It must also be acknowledged that the present study did not include an analysis of the possible impact of ecological factors like family, neighborhood, or community on the parent prenatal LOC/child outcomes. Bronfenbrenner (2015) has pointed out the limiting and facilitating effect ecological factors can have on parent, child interaction.

## Conclusion

In the present study, children’s outcome data were gathered from independent raters from outside the home. The children were in school and rated by teachers, not family members, thereby eliminating the potential for parent bias to affect the results. Teachers’ judgments of children’s outcomes were consistent with those gathered from parents’ ratings obtained earlier in life; greater presence of prenatal externality was associated with negative child outcomes in children 8 and 11 years later.

## Author Contributions

SN had the idea; JG planned the analyses; GE and SG undertook the statistical analyses; JG and SN wrote the first draft. All authors contributed equally to writing later drafts, checking, and editing.

## Conflict of Interest Statement

The authors declare that the research was conducted in the absence of any commercial or financial relationships that could be construed as a potential conflict of interest.

## Abbreviations

ALSPAC: Avon longitudinal study of parents and children
ANSIE: adult Nowicki–Strickland internal–external locus of control scale
LOC: locus of control
SDQ: strengths and difficulties’ questionnaire
