# Supplementary material for: Parental External Locus of Control in Pregnancy Is Associated with Subsequent Teacher Ratings of Negative Behavior in Primary School: Findings from a British Birth Cohort
Source: Front Psychol. 2018 Feb 9;9:120. doi: 10.3389/fpsyg.2018.00120 (PMC5811656; doi:10.3389/fpsyg.2018.00120)
Supplement: Supplementary file 1 [file Table_1.DOCX]

**Supplementary Tables for SDQ paper I1**

Supplementary Table 1. Availability of teacher completed SDQ for children whose mothers completed the LOC assessment in pregnancy.

| **Maternal Variable** | **SDQ Year 3** | | **SDQ Year 6** | |
| --- | --- | --- | --- | --- |
|  | **Available** | **Not Available** | **Available** | **Not**  **Available** |
| Maternal age at conception |  |  |  |  |
| < 25 | 1,337 (24.3%) | 1,968 (28.3%) | 1,689 (26.1%) | 1,656 (27.1%) |
| 25 – 29 | 2,370 (41.9%) | 2,736 (39.4%) | 2,689 (41.6%) | 2,408 (39.4%) |
| ≥ 30 | 1,908 (33.7%) | 2,242 (32.3%) | 2,097 (32.3%) | 2,053 (33.6%) |
| P using χ² |  | **<0.001** |  | **0.037** |
|  |  |  |  |  |
| Housing tenure |  |  |  |  |
| Owned/mortgaged | 4,431 (80.7%) | 4,764 (70.8%) | 4,904 (78.1%) | 4,291 (72.3%) |
| Public housing | 570 (10.4%) | 1,014 (15.1%) | 776 (12.3%) | 808 (13.6%) |
| All other | 487 (8.9%) | 948 (14.1%) | 601 (9.6%) | 834 (14.1%) |
| P using χ² |  | **<0.001** |  | **<0.001** |
|  |  |  |  |  |
| Crowding index (people/room) |  |  |  |  |
| ≤ 0.5 | 2,400 (44.2%) | 2,744 (41.6%) | 2,659 (42.9%) | 2.485 (42.7%) |
| >0.5-0.75 | 1,770 (32.6%) | 2,025 (30.7%) | 1,993 (32.2%) | 1,802 (30.9%) |
| >0.75-1.0 | 984 (18.1%) | 1,363 (20.6%) | 1,193 (19.2%) | 1,154 (19.8%) |
| >1.0 | 270 (5.0%) | 469 (7.1%) | 353 (5.7%) | 386 (6.6%) |
| P using χ² |  | **<0.001** |  | 0.103 |
|  |  |  |  |  |
| Parity |  |  |  |  |
| 0 | 2,429 (44.7%) | 2,997 (45.0%) | 2,792 (44.9%) | 2.634 (44.9%) |
| 1 | 1,956 (36.0%) | 2,321 (34.9%) | 2,192 (35.2%) | 2,085 (35.6%) |
| ≥ 2 | 1,045 (19.2%) | 1,337 (20.1%) | 1,238 (19.9%) | 1,144 (19.5%) |
| P using χ² |  | 0.324 |  | 0.851 |
|  |  |  |  |  |
| Maternal education level |  |  |  |  |
| <O Level | 1,518 (28.2%) | 1,902 (29.4%) | 1,790 (29.1%) | 1,630 (28.6%) |
| O Level | 1,916 (35.5%) | 2,234 (34.6%) | 2,239 (36.4%) | 1,911 (33.5%) |
| >O Level | 1,955 (36.3%) | 2,326 (36.0%) | 2,116 (34.4%) | 2,165 (37.9%) |
| P using χ² |  | 0.286 |  | **<0.001** |
|  |  |  |  |  |
| Difficulty affording food |  |  |  |  |
| Yes | 1,188 (22.6%) | 1,550 (24.4%) | 1,399 (23.3%) | 1,339 (24.0%) |
| No | 4,066 (77.4%) | 4,792 (75.6%) | 4,610 (76.7%) | 4,248 (76.0%) |
| P using χ² |  | **0.021** |  | 0.386 |
|  |  |  |  |  |
| Smoked mid-pregnancy |  |  |  |  |
| Yes | 991 (17.9%) | 1,333 (19.7%) | 1,178 (18.6%) | 1,146 (19.3%) |
| No | 4,552 (82.1%) | 5,420 (80.3%) | 5,169 (81.4%) | 4,803 (80.7%) |
| P using χ² |  | **0.009** |  | 0.319 |
|  |  |  |  |  |
| Binge drinking |  |  |  |  |
| None | 4,619 (84.2%) | 5,522 (82.8%) | 5,262 (83.7%) | 4,879 (83.1%) |
| Any | 867 (15.8%) | 1,146 (17.2%) | 1,022 (16.3%) | 991 (16.9%) |
| P using χ² |  | **0.041** |  | 0.359 |
|  |  |  |  |  |
| Depression score at 18wks |  |  |  |  |
| < 12 | 4,333 (83.7%) | 5,191 (82.3%) | 4,938 (82.9%) | 4,586 (83.0%) |
| ≥ 12 | 843 (16.3%) | 1,113 (17.7%) | 1,015 (17.1%) | 941 (17.0%) |
| P using χ² |  | 0.052 |  | 0.972 |
|  |  |  |  |  |
| Locus of control score |  |  |  |  |
| External | 2,512 (44.4%) | 3,205 (45.9%) | 2,927 (45.1%) | 2,790 (45.4%) |
| Internal | 3,143 (55.6%) | 3,770 (54.1%) | 3,557 (54.9%) | 3,356 (54.6%) |
| P using χ² |  | 0.086 |  | 0.775 |

# Supplementary Table 2. Correlation matrix showing parents’ Locus of Control score in pregnancy and child’s behaviour in school at school years 3 & 6 (N = 2359)

|  | **Mat LOC** | **Pat LOC** | **ProS Y3** | **Hyp Y3** | **EmD Y3** | **CD Y3** | **PP Y3** | **TotD Y3** | **ProS Y6** | **Hyp Y6** | **EmD Y6** | **CD Y6** | **PP Y6** |
| --- | --- | --- | --- | --- | --- | --- | --- | --- | --- | --- | --- | --- | --- |
| **Pat LOC** | 0.3163 | 1 |  |  |  |  |  |  |  |  |  |  |  |
| **ProS Y3** | -0.0795 | -0.0342 | 1 |  |  |  |  |  |  |  |  |  |  |
| **Hyp Y3** | 0.1531 | 0.1151 | -0.5791 | 1 |  |  |  |  |  |  |  |  |  |
| **EmD Y3** | 0.083 | 0.0526 | -0.1591 | 0.1892 | 1 |  |  |  |  |  |  |  |  |
| **CD Y3** | 0.1018 | 0.0747 | -0.5188 | 0.5878 | 0.1361 | 1 |  |  |  |  |  |  |  |
| **PP Y3** | 0.0637 | 0.029 | -0.4356 | 0.3722 | 0.415 | 0.3575 | 1 |  |  |  |  |  |  |
| **TotD Y3** | 0.1475 | 0.1009 | -0.5968 | 0.804 | 0.6124 | 0.6825 | 0.7324 | 1 |  |  |  |  |  |
| **ProS Y6** | -0.0453 | -0.0448 | 0.3344 | -0.3348 | -0.0512 | -0.3058 | -0.2118 | -0.3191 | 1 |  |  |  |  |
| **Hyp Y6** | 0.1429 | 0.1239 | -0.3539 | 0.5796 | 0.1024 | 0.4208 | 0.2521 | 0.4944 | -0.543 | 1 |  |  |  |
| **EmD Y6** | 0.0638 | 0.0306 | -0.1237 | 0.1583 | 0.3143 | 0.1003 | 0.2443 | 0.2895 | -0.1857 | 0.2336 | 1 |  |  |
| **CD Y6** | 0.079 | 0.0996 | -0.2918 | 0.3794 | 0.0433 | 0.4607 | 0.2217 | 0.3776 | -0.5411 | 0.5868 | 0.1722 | 1 |  |
| **PP Y6** | 0.0494 | 0.0347 | -0.2418 | 0.2537 | 0.2069 | 0.2332 | 0.4357 | 0.3903 | -0.4233 | 0.3035 | 0.5028 | 0.3604 | 1 |
| **TotD Y6** | 0.1231 | 0.1027 | -0.3559 | 0.4955 | 0.2353 | 0.4167 | 0.3997 | 0.548 | -0.5856 | 0.7792 | 0.6627 | 0.6921 | 0.7371 |

| Mat = Maternal | Hyp = Hyperactivity |
| --- | --- |
| Pat = Paternal | EmD = Emotional difficulties |
| LOC = Locus of Control | PP = Peer problems |
| ProS = Prosocial behavior | TotD = Total behavioural difficulties |
| CD = Conduct difficulties | Y3 = Year 3 |
|  | Y6 = Year 6 |

Supplementary Table 3: Frequencies within the four categories of parental locus of control orientation

|  | **M.Ex. F.Ex** | **M.Ex. F.In** | **M.In. F.Ex** | **M.In. F.In** |
| --- | --- | --- | --- | --- |
|  |  |  |  |  |
| Maternal age at conception |  |  |  |  |
| < 25 | 40.4% (906) | 23.1% (334) | 26.8% (485) | 13.3% (422) |
| 25 – 29 | 37.6% (843) | 42.8% (618) | 41.8% (757) | 41.9% (1334) |
| ≥ 30 | 22.0% (494) | 34.1% (493) | 31.5% (571) | 44.8% (1429) |
| P using χ² |  |  |  |  |
|  |  |  |  |  |
| Housing tenure |  |  |  |  |
| Owned/mortgaged | 60.3% (1310) | 81.6% (1138) | 79.6% (1418) | 89.6% (2797) |
| Public housing | 24.6% (534) | 9.0% (126) | 11.0% (196) | 2.3% (72) |
| All other | 15.1% (328) | 9.4% (131) | 9.4% (168) | 8.1% (252) |
| P using χ² |  |  |  |  |
|  |  |  |  |  |
| Crowding index (people/room) |  |  |  |  |
| ≤ 0.5 | 29.2% (621) | 43.5% (598) | 45.8% (807) | 58.3% (1804) |
| >0.5-0.75 | 32.2% (685) | 34.0% (468) | 30.0% (528) | 28.7% (887) |
| >0.75-1.0 | 28.1% (598) | 18.2% (251) | 19.0% (335) | 11.0% (341) |
| >1.0 | 10.4% (222) | 4.3% (59) | 5.2% (92) | 2.0% (62) |
| P using χ² |  |  |  |  |
|  |  |  |  |  |
| Parity |  |  |  |  |
| 0 | 43.3% (934) | 45.3% (626) | 48.1% (849) | 50.2% (1551) |
| 1 | 33.8% (729) | 34.1% (471) | 34.5% (610) | 34.5% (1067) |
| ≥ 2 | 22.9% (494) | 20.7% (285) | 17.3% (307) | 15.3% (474) |
| P using χ² |  |  |  |  |
|  |  |  |  |  |
| Maternal education level |  |  |  |  |
| <O Level | 47.4% (986) | 34.6% (476) | 21.9% (382) | 11.5% (360) |
| O Level | 37.3% (775) | 37.1% (510) | 39.9% (695) | 30.6% (952) |
| >O Level | 15.3% (319) | 28.3% (389) | 38.2% (666) | 57.9% (1802) |
| P using χ² |  |  |  |  |
|  |  |  |  |  |
| Paternal education level |  |  |  |  |
| <O Level | 49.2% (975) | 20.0% (257) | 34.7% (572) | 11.3% (336) |
| O Level | 23.5% (466) | 25.0% (321) | 25.0% (412) | 20.7% (613) |
| >O Level | 27.3% (541) | 55.0% (705) | 40.3% (665) | 68.1% (2019) |
| P using χ² |  |  |  |  |
|  |  |  |  |  |
| Difficulty affording food |  |  |  |  |
| Yes | 34.2% (702) | 22.5% (303) | 22.2% (379) | 12.7% (383) |
| No | 65.7% (1347) | 77.5% (1044) | 77.8% (1331) | 87.4% (2657) |
| P using χ² |  |  |  |  |
|  |  |  |  |  |
| Mother smoked mid-pregnancy |  |  |  |  |
| Yes | 28.9% (632) | 18.5% (262) | 16.4% (292) | 7.3% (231) |
| No | 71.1% (1554) | 81.5% (1157) | 83.6% (1491) | 92.7% (2918) |
| P using χ² |  |  |  |  |
|  |  |  |  |  |
|  |  |  |  |  |
| Father smoked mid-pregnancy |  |  |  |  |
| Yes | 41.4% (711) | 24.8% (292) | 30.9% (452) | 15.4% (420) |
| No | 58.6% (1008) | 75.2% (886) | 69.1% (1012) | 84.6% (2310) |
| P using χ² |  |  |  |  |
|  |  |  |  |  |
| Maternal binge drinking mid-pregnancy |  |  |  |  |
| None | 80.5% (1742) | 82.9% (1164) | 83.7% (1481) | 89.0% (2784) |
| Any | 19.5% (421) | 17.1% (240) | 16.3% (289) | 11.0% (343) |
| P using χ² |  |  |  |  |
|  |  |  |  |  |
| Paternal binge drinking mid-pregnancy |  |  |  |  |
| None | 20.8% (416) | 16.4% (213) | 16.8% (277) | 17.9% (533) |
| Any | 79.2% (1582) | 83.6% (1301) | 83.2% (1367) | 82.1% (2972) |
| P using χ² |  |  |  |  |
|  |  |  |  |  |
| Maternal depression score at 18wks (EPDS) |  |  |  |  |
| Mean (SD) | 7.83 (5.11) | 7.11 (4.81) | 6.43 (4.49) | 5.82 (4.28) |
| P using χ² |  |  |  |  |
|  |  |  |  |  |
| Paternal depression score at 18wks (EPDS) |  |  |  |  |
| Mean (SD) | 4.64 (4.28) | 3.83 (3.53) | 4.32 (3.86) | 3.60 (3.43) |
| P using χ² |  |  |  |  |
|  |  |  |  |  |
| Maternal LOC score |  |  |  |  |
| Mean (SD) | 6.35 (1.36) | 5.95 (1.17) | 3.03 (1.04) | 2.60 (1.15) |
| P using χ² |  |  |  |  |
|  |  |  |  |  |
| Paternal LOC score |  |  |  |  |
| Mean (SD) | 5.87 (1.69) | 1.92 (0.94) | 5.30 (1.46) | 1.79 (0.98) |
| P using χ² |  |  |  |  |
|  |  |  |  |  |

N.B. Paternal binge drinking is ‘in last 2m of pregnancy’ as opposed to mid-preg for mum

Supplementary Table 4. Proportion in the lowest 15% of teacher ratings of child behaviour using SDQ according to the LOC orientation of the child’s parents as measured in pregnancy. [Asterisks indicate differences between the pairs of Father orientation as *P<0.05; **P<0.01; ***P<0.001]

| **Child behavior** | **M.Ex. F.Ex** | **M.Ex. F.In** | **M.In. F.Ex** | **M.In.F.In** |
| --- | --- | --- | --- | --- |
|  |  |  |  |  |
| Hyperactivity |  |  |  |  |
| Year 3 | 18.9%[186] | 14.2%[98]*** | 12.9%[106] | 10.6%[158]* |
|  |  |  |  |  |
| Year 6 | 17.8%[210] | 10.0%[75]*** | 11.8%[112] | 7.8[127]*** |
|  |  |  |  |  |
|  |  |  |  |  |
| Emotional Problems |  |  |  |  |
| Year 3 | 16.3%[160] | 14.9%[103] | 12.8%[105] | 11.0%[163] |
|  |  |  |  |  |
| Year 6 | 15.2%[179] | 12.7%[95] | 12.3%[117] | 11.0%[179] |
|  |  |  |  |  |
|  |  |  |  |  |
| Conduct problems |  |  |  |  |
| Year 3 | 12.4%[122] | 8.9[61]* | 7.8%[64] | 6.0%[89] |
|  |  |  |  |  |
| Year 6 | 15.2%[179] | 7.9%[59]*** | 11.1%[106] | 7.3%[119]*** |
|  |  |  |  |  |
|  |  |  |  |  |
| Peer Problems |  |  |  |  |
| Year 3 | 11.7%[115] | 11.9%[82] | 10.1%[83] | 10.0%[149] |
|  |  |  |  |  |
| Year 6 | 12.1%[143] | 9.2%[69]* | 13.1%[125] | 10.7%[174] |
|  |  |  |  |  |
|  |  |  |  |  |
| Total difficulties |  |  |  |  |
| Year 3 | 16.6%[163] | 13.2[91]* | 10.3%[85] | 8.6%[128]* |
|  |  |  |  |  |
| Year 6 | 15.7%[185] | 9.5[71]*** | 11.5%[109] | 8.9%[144]** |
|  |  |  |  |  |

Supplementary Table 5: The change in the mean of the child’s behavior score according to the numbers of parents who have an external orientation during pregnancy [the mean differences [MD] are per increase of one external parent].

| **SDQ** | **Unadjusted** | | **Adjusted** | |
| --- | --- | --- | --- | --- |
|  | **MD [95% CI]** | **P** | **MD [95% CI]** | **P** |
|  |  |  |  |  |
| Prosocial |  |  |  |  |
| Year 3 | **-0.14 [-0.24,-0.05]** | **0.003** | -0.07 [+0.03, -1.16] | 0.145 |
| Year 6 | **-0.16 [-0.25,-0.08]** | **<0.001** | **-0.13 [-0.23,-0.04]** | **0.005** |
|  |  |  |  |  |
| Hyperactivity |  |  |  |  |
| Year 3 | **0.45 [0.35,0.56]** | **<0.001** | **0.38 [0.27,0.49]** | **<0.001** |
| Year 6 | **0.54 [0.45,0.63]** | **<0.001** | **0.44 [0.35,0.54]** | **<0.001** |
|  |  |  |  |  |
| Emotional symptoms |  |  |  |  |
| Year 3 | **0.17 [0.10,0.25]** | **<0.001** | **0.14 [0.06,0.23]** | **0.001** |
| Year 6 | **0.15 [0.08,0.22]** | **<0.001** | **0.12 [0.04,0.20]** | **0.002** |
|  |  |  |  |  |
| Conduct problems |  |  |  |  |
| Year 3 | **0.17 [0.11,0.22]** | **<0.001** | **0.11 [0.05,0.16]** | **<0.001** |
| Year 6 | **0.23 [0.17,0.28]** | **<0.001** | **0.16 [0.11,0.22]** | **<0.001** |
|  |  |  |  |  |
| Peer problems |  |  |  |  |
| Year 3 | **0.11 [0.05,0.18]** | **0.001** | **0.11 [0.03,0.18]** | **0.006** |
| Year 6 | **0.08 [0.01,0.15]** | **0.020** | 0.07 [-0.01,0.14] | 0.068 |
|  |  |  |  |  |
| Total difficulties |  |  |  |  |
| Year 3 | **0.92 [0.71,1.14]** | **<0.001** | **0.74 [0.51,0.97]** | **<0.001** |
| Year 6 | **1.00 [0.79,1.21]** | **<0.001** | **0.80 [0.58,1.02]** | **<0.001** |

*Adjusted for maternal age, residence in public rented housing and child’s sex
